# Effect of Feeding Rate on Growth Performance and Waste Reduction Efficiency of Black Soldier Fly Larvae (Diptera: Stratiomyidae)

**DOI:** 10.21315/tlsr2022.33.1.11

**Published:** 2022-03-31

**Authors:** Moo Chee Yuan, Hadura Abu Hasan

**Affiliations:** 1 School of Biological Sciences, Universiti Sains Malaysia, 11800 USM Pulau Pinang, Malaysia; 2 Vector Control Research Unit, School of Biological Sciences, Universiti Sains Malaysia, 11800 USM Pulau Pinang, Malaysia

**Keywords:** Black Soldier Fly Larvae, Feeding Rate, Growth Performance, *Hermetia illucens*, Organic Waste, Waste Reduction, Larva Lalat Askar Hitam, Kadar Pemakanan, Prestasi Pertumbuhan, *Hermetia illucens*, Sisa Organik, Pengurangan Sisa

## Abstract

Malaysia like many other developing countries is facing the challenge of poor waste management. This research was conducted to determine the effect of black soldier fly (BSF) larvae in decomposing food waste, palm oil waste, fish waste and yard waste. The development time and waste reduction efficiency of four different organic materials were evaluated. In this study, BSF larvae were fed with all four types of waste at five feeding rates of 0.25, 0.50, 1.00, 1.50 and 2.00 g larva^−1^ day^−1^ with three replicates per feeding rate until the larvae reached the pre-pupae stage. During the study, larval development time, larval mortality, pre-pupae weight and waste reduction indexes (WRI) were determined. Food waste and yard waste achieved the highest WRI of 4.43 ± 0.06 and 0.71 ± 0.01, respectively at the feeding rate of 0.50 g larva^−1^ day^−1^ while palm oil waste and fish waste attained the highest WRI values at feeding rates of 1.00 g larva^−1^ day^−1^ (1.89 ± 0.02) and 0.25 g larva^−1^ day^−1^ (3.75 ± 0.24), respectively. The results showed that both variables significantly influenced the bioconversion process, but waste reduction efficiency was the most influential element.

## Abstract (fr)

Malaysia seperti kebanyakan negara membangun yang lain menghadapi cabaran pengurusan sampah yang kurang baik. Penyelidikan ini dilakukan untuk mengetahui kesan larva lalat askar hitam dalam menguraikan sisa makanan, sisa kelapa sawit, sisa ikan dan sisa halaman. Masa perkembangan dan kecekapan pengurangan sisa empat bahan organik telah dinilai. Dalam kajian ini, larva lalat askar hitam diberi makan dengan empat jenis sisa pada lima kadar pemakanan iaitu 0.25, 0.50, 1.00, 1.50 dan 2.00 g larva^−1^ hari^−1^ dengan tiga ulangan setiap kadar pemakanan sehingga larva mencapai peringkat pra-pupa. Semasa kajian, masa perkembangan larva, kematian larva, berat badan pra-pupa dan indeks pengurangan sisa ditentukan. Sisa makanan dan sisa halaman mencapai indeks pengurangan sisa tertinggi iaitu 4.43 ± 0.06 dan 0.71 ± 0.01, masing-masing pada kadar makan 0.50 g larva^−1^ hari^−1^ sementara sisa kelapa sawit dan sisa ikan mencapai nilai indeks pengurangan sisa tertinggi pada kadar pemakanan 1.00 g larva^−1^ hari^−1^ (1.89 ± 0.02) dan 0.25 g larva^−1^ hari^−1^ (3.75 ± 0.24), masing-masing. Hasil kajian menunjukkan bahawa kedua-dua pemboleh ubah mempengaruhi proses bio-penukaran secara signifikan, tetapi kecekapan pengurangan sisa merupakan elemen yang paling berpengaruh.

**Highlight**

- The effects of black soldier fly larvae in decomposing food waste, palm oil waste, fish waste, and yard waste were recorded as development time, pre-pupae weight, mortality, and waste reduction efficiency (waste reduction index).
- Food waste showed the highest means of waste reduction index (WRI), while the lowest WRI was recorded from yard waste.
- Low mortality of black soldier fly larvae was observed in food waste, palm oil waste, and fish waste (feeding substrates).

## INTRODUCTION

Increasing population, rapid economic growth, urbanisation and industrialisation had caused the accumulation of solid waste in most developing countries. Diversity in consumption patterns and insufficient knowledge to implement integrated programmes that assimilate environmental and cleaner technologies have increased the global concern towards solid waste management (Diener *et al*. 2009). Creating sustainable solid waste management solutions is the mission of all countries around the world. Composting is a controlled biological process that breaks organic material into carbon dioxide, water, minerals and stabilised organic matter and reduces solid waste volume (Saheri *et al*. 2009). Decomposing municipal solid waste (MSW) with black soldier fly (BSF), *Hermetia illucens* (L.) demonstrated the potential use of this species in waste management systems especially in the bioconversion of food waste (Diener *et al*. 2009; 2011; 2015; Jucker *et al*. 2017; Gold *et al*. 2018). The BSF is a non-pest insect distributed throughout the world. This fly species occurs naturally in areas with abundant organic materials (Singh & Kumari 2019). BSF larvae can consume a wide range of organic materials and have the potential to use in waste management (Nguyen *et al*. 2015).

The organic composition of solid waste generated in the least developed countries such as Bangladesh or Pakistan is higher than 65%, whilst in developed countries such as Japan and the Republic of Korea, the percentage is below 30% (Mmereki *et al*. 2016). A similar situation in which the composition of solid waste dominated by organic waste is happening in Malaysia as the largest portion of MSW is food waste. The utilisation of BSF for treating various organic waste has been greatly studied in many countries. BSF larval performance varies with the quality and type of feed substrate (Lalander *et al*. 2019; Rehman, Rehman *et al*. 2017; Liu, Awasthi, *et al*. 2021).

Furthermore, the study not only focuses on homogenous organic material which has uniform composition and properties such as manure of domestic animals but also other organic waste such as food waste, household waste or faecal sludge which has mixed composition and properties as a feed source (Spranghers *et al*. 2017; Nyakeri *et al*. 2017b; Kawasaki *et al*. 2019). Yet most municipal organic solid waste has a mixed composition. Diener *et al*. (2011) conducted a medium-scale experiment in Costa Rica to explore the benefits and limitations of the BSF technology. A waste reduction ranging from 65.5% to 78.9% was achieved in that study but the composition of municipal organic waste was not further explained. The research should head more on the utilisation of BSF larvae to decompose agricultural waste, food industry processing waste and landscaping waste. These wastes create significant challenges towards optimising the most favourable conditions for the evolution and development of BSF larvae in tropical and sub-tropical countries.

The bioconversion technology using the BSF larvae in treating organic solid waste is becoming progressively popular around the world, especially in developing countries. The proposed feeding rates for BSF larvae were documented by previous studies as 100 mg larva^−1^ day^−1^ (Diener *et al*. 2009), 163 mg larva^−1^ day^−1^ (dry base) (Paz *et al*. 2015) and 200 mg larva^−1^ day^−1^ (Permana *et al*. 2018). These feeding rates were suggested to achieve optimum nutrient demand and digestion process. Although standard food quantity has been suggested for the BSF larvae, the impact on bioconversion rate and waste reduction efficiency of the non-standard quantity of food supply are not discussed in detail.

In this study, different feeding rates were evaluated to determine the effects of BSF in decomposing food waste, palm oil waste, fish waste and yard waste. The effects were documented in terms of insect growth (development time, pre-pupae weight and mortality) and a waste reduction efficiency (waste reduction index). Ultimately, we expect to find significant insights for the small and large-scale industry when applying BSF larvae for the bioconversion process in waste management and animal feed production.

## MATERIALS AND METHODS

### Black Soldier Fly Larvae

The eggs of BSF were collected from trapping bins (43 cm diameter × 50 cm height). The trapping bins consisted of attractant media and wooden blocks (29 cm × 3 cm × 1 cm), spaced by cable ties and held by elastic bands. Attractant media in trapping bins composed mainly a mixture of coconut waste and effective microorganisms (EM) with a ratio of 1:1. The media was fermented for four days before placing the trapping bins in the field (Hasan & Dina 2019). The eggs were collected 24 h–48 h after the placement of the trapping bins. Upon hatching, larvae were transferred to plastic containers (20 cm × 20 cm × 10 cm) and reared with attractant media. The containers were covered using mesh nets and lids to avoid larvae escaping and the larvae were counted after 4–5 days. In each of the treatments, 100 BSF larvae were used for the bioconversion of different organic waste. The initial larval weight was recorded before the introduction to the waste matrix. The age of the larvae used in the experiment was five to six days after hatching. The experiment was carried out at 26°C temperature, 60%–70% relative humidity and 12 h photoperiod in insectarium at the School of Biological Sciences, Universiti Sains Malaysia (USM), Pulau Pinang, Malaysia.

### Organic Waste

Four types of organic solid waste were collected to assess the ability of BSF larvae in digesting and decomposing the waste. The wastes comprised of food waste from campus cafeteria, palm oil waste from Malpom Industry Sdn. Bhd., fish waste from Protigam Sdn. Bhd. and yard waste from Taman Floral, School of Biological Sciences, USM. Food waste and yard waste were chosen as they contribute to a major part of organic waste proportion in the MSW composition of Malaysia. Meanwhile, palm oil decanter cake was selected as one of the organic solid wastes tested in this experiment as it is always dumped beside the mill after the extraction process of palm oil due to the lack of proper management systems. Fish waste was used as it was the common waste of the canned food production industry that can be found in the landfill. To assess waste reduction capacity by BSF larvae, a measured weight of organic wastes was used for each treatment.

### Collection of Waste

Food waste was collected daily from the campus cafeteria by placing a waste bin at Fajar Harapan Cafeteria. All the food waste was gathered daily in black polythene bags and taken to the laboratory for the segregation process. This secures the food waste from fungus or bacteria decomposition and flies’ infestation. Conversely, palm oil waste, fish waste and yard waste were collected in bulk from respective sources. Palm oil waste, the palm oil decanter cake was collected from Malpom Industries Sdn. Bhd., fish waste, the mackerel fish’s head was collected from Protigam Sdn. Bhd. and yard waste, the mango tree leaves were collected from Taman Floral, School of Biological Sciences, USM. Palm oil waste was placed in a sealed bucket, mackerel fish’s head was placed in a black waste bin and the yard waste was kept in a plastic container. All the wastes were transported back to the laboratory for further processing and segregation.

### Processing and Segregating Waste

Unwanted wastes such straws, plastic cups, tins and tissue were separated and removed from the food waste. Then, indigestible parts like bones inside meats and hard seeds from vegetables were removed as well. After that, the food waste was ground with a chopper (ITATA, 250W and 2 L) to get a fine texture before introducing them to the BSF larvae. This was to increase the surface area to be fed by BSF larvae. The palm oil waste was packed in a zipper bag (3.78 L) and placed in a freezer to maintain the freshness of the waste. Cooked fish waste (Mackerel) was ground with a chopper (ITATA, 250W and 2 L). The fine paste of fish waste was packed in a zipper bag (3.78 litres) and stored in a freezer to ensure the freshness of the waste. Dry mango tree leaves were shredded into powder form with an industrial grinder (300-micron mesh) at Soil Science Laboratory, School of Biological Sciences, USM. The yard waste powder was packed in a zipper bag (3.78 L). The yard waste powder was mixed with distilled water in one to three ratios during the experiment. Palm oil waste and fish waste were thawed to room temperature before inoculating to BSF larvae.

### Experimental Design

Food waste, palm oil waste, fish waste and yard waste were used as treatments. Five feeding rates were employed on each type of waste. The feeding rate conducted in this study comprised 0.25, 0.50, 1.00, 1.50 and 2.00 g larva^−1^ day^−1^ with three replicates per feeding rate. The selected quantities of waste were different from the amount suggested by Diener *et al*. (2009), Paz *et al. (*2015) and Permana *et al*. (2018). A total of 20 transparent plastic containers (20 cm × 20 cm × 10 cm) with lids were used, five replicates for each feeding rate in one single set of experiments. Each feeding container contained the designated waste quantity and 100 of five-six days-old BSF larvae. Every container was covered with mosquito mesh to avoid oviposition by other flies. Twelve holes (diameter, 0.5 cm) were punctured on the lid to ensure air circulation. All the experimental setups were carried out in an insectarium at a constant temperature, humidity and photoperiod as mentioned above.

For each treatment, hundred BSF larvae were weighed and recorded before inoculating them into the waste matrix. The weight of each designated waste quantity was also measured and recorded before being fed to the BSF larvae. The feeding process was discontinued after 50% of larvae reached the pre-pupae stage, indicated by a colour change from creamy white to dark brown. The residue (excretory product) and the pre-pupae weight were measured at the end of the experiment. All the data recorded was in form of wet weight measurements. The larval mortality was determined by using the formula employed by Leong *et al*. (2016), while the efficiency of the BSF larvae to consume and reduce organic matter content in the fed substrates was determined by calculation of waste reduction index (WRI) as described previously by Diener *et al*. (2009).

### Determination of variables

#### Larval mortality (LM)

Larvae mortality is the number of larvae dead during experiment or number of larvae that does not transform into pre-pupae.


$$LM\;(\%)=\frac{\left[\text{Initial number of larvae }(100)-(\text{Final number of larvae}+\text{Number of pre-pupae})\right]}{\text{Initial number of larvae}\times 100}$$

#### Waste reduction index (WRI)

Waste reduction index is the time taken by larvae required to reduce specific amount of waste. High WRI values indicate good reduction efficiency.

#### Decomposition rate of waste (D)


$$D=W-\frac{R}{W}$$

*W* = Total amount of organic waste applied (g)
*R* = Total amount of residue produced (g)

$$WRI=\frac{D}{t}\times 100$$

*t* = experiment and factor 100 give the index a practical value.

### Statistical Analysis

IBM SPSS Statistics version 24 program was used to perform the statistical analysis. Tukey’s HSD (honestly significant difference) post-hoc test with a 95% confidence interval was used to test the significant differences in the means (*p* < 0.05). One-way ANOVA, with subsequent Tukey’s HSD tests, was conducted to determine the differences in the means development time, larval mortality, larvae/pre-pupae wet weight and WRI among various feeding rates of all four organic solid waste. The effects of various feeding rates and different types of waste on pre-pupae weight and WRI were analysed using two-way ANOVA.

## RESULTS

### Insect Growth and Waste Reduction Efficiency

Insect growth was interpreted as pre-pupae weight, development time and mortality rate of BSF larvae. Waste reduction efficiency was obtained by calculating the decomposition rate of waste (D) and recorded as waste reduction index (WRI).

### Insect Growth (Larval Weight, Development Time and Larval Mortality Rate)

#### Food waste

The pre-pupae weight showed several trends, whereby it increased from feeding rates 0.25 g to 1.00 g larva^−1^ day^−1^, decreased from 1.00 g to 1.50 g larva^−1^ day^−1^ before increasing again to 2.00 g larva^−1^ day^−1^ as depicted in Table 1. The highest pre-pupae weight was 12.73 ± 0.24 g (12 days) at a feeding rate of 1.00 g larva^−1^ day^−1^ (Table 1). The development time of larvae showed a decreasing trend as the feeding rate using food waste increases. The longest development time was 20 days with a feeding rate of 0.25 g larva^−1^ day^−1^ while the shortest development time was 11 days at 2.00 g larva^−1^ day^−1^. The lowest mortality rate was 0.67 ± 0.67% at feeding rates 1.00 g and 1.50 g larva^−1^ day^−1^ (Table 1). However, the differences in mortality rate among the feeding rates were not significant (Tukey’s HSD; *p* = 0.32).

#### Palm oil waste

The highest pre-pupae weight of 11.90 ± 0.38 g (13 days) was obtained at a feeding rate of 1.50 g larva^−1^ day^−1^ (Table 2) and significantly higher than the other feeding rates (Tukey’s HSD; *p* < 0.05). Development time decreased as the feeding rate using palm oil waste increased. The longest development time was 19 days with a feeding rate of 0.25 g larva^−1^ day^−1^ while the shortest development time was 12 days at 2.00 g larva^−1^ day^−1^ (Table 2). The feeding rates of 1.00 g and 1.50 g larva^−1^ day^−1^ both showed the lowest mortality rates at 2.67 ± 1.53%, there was no significant difference in mortality rate among all feeding rates (Tukey’s HSD; *p* = 0.68).

#### Fish waste

The pre-pupae weight showed an increasing trend from 0.25 g to 1.50 g larva^−1^ day^−1^ feeding rate. The highest pre-pupae weight obtained was 5.69 ± 0.06 g (12 days) at a feeding rate of 1.50 g larva^−1^ day^−1^. The shortest development time (10 days) was recorded at a feeding rate of 2.00 g larva^−1^ day^−1^ (Table 3). No significant difference in larval mortality against feeding rates was observed in fish waste (Tukey’s HSD; *p* = 0.22).

#### Yard waste

**Figure 1:**
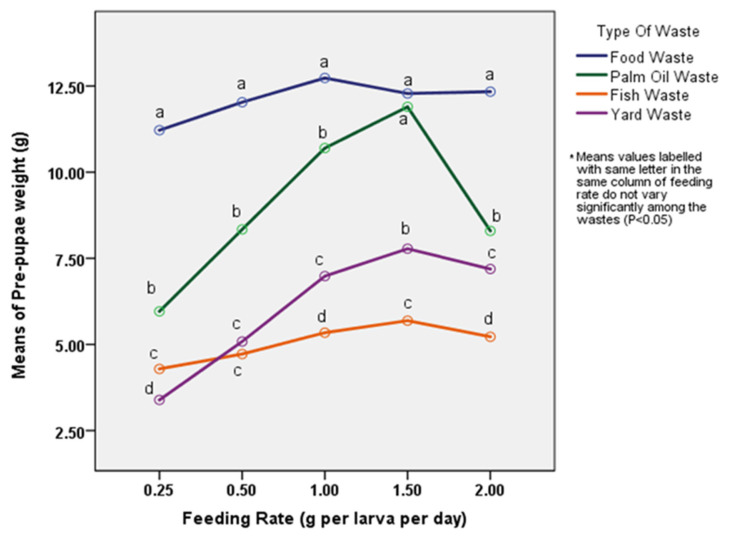
Pre-pupae weight of BSF larvae fed on different amounts of organic wastes.

The larvae/pre-pupae weight showed an increasing trend from 0.25 g to 1.50 g larva^−1^ day^−1^ feeding rate before decreasing from 1.50 g to 2.00 g larva^−1^ day^−1^ feeding rate. The highest pre-pupae/larvae weight gained was 7.78 ± 0.14 g after a development time of 56 days. The lowest and highest larval mortality recorded were 6.33 ± 0.88 (1.00 g larva^−1^ day^−1^) and 14.00 ± 0.58 (1.50 g larva^−1^ day^−1^) respectively (Table 4). There were significant differences in larvae mortality among feeding rates (Tukey’s HSD; *p* < 0.05). Mean of pre-pupae weight on four different types of waste (food waste, palm oil waste, fish waste and yard waste) against feeding rate (0.25 g, 0.50 g, 1.00 g, 1.50 g and 2.00 g larva^−1^ day^−1^) were compared in Fig. 1. The interaction between feeding rate and type of waste was significantly different (F = 26.05, *p* < 0.05).

### Waste Reduction Index

**Figure 2:**
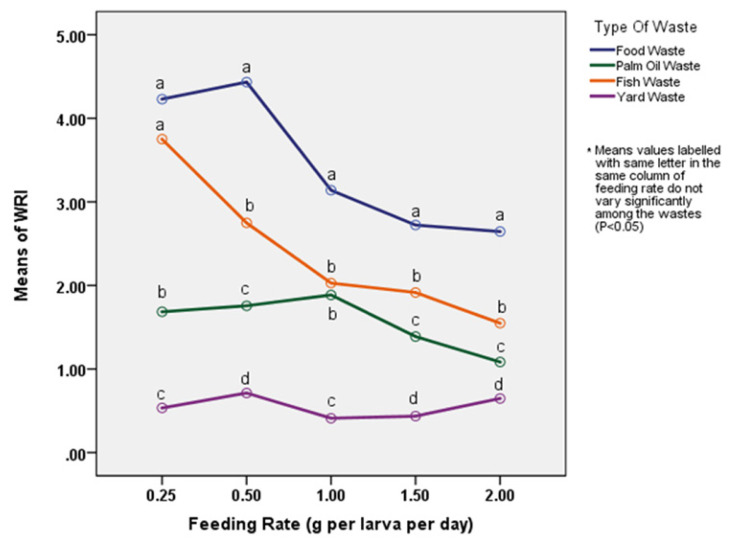
Waste reduction index of BSF larvae fed on different amounts of organic wastes.

Food waste showed the highest estimated marginal means of WRI at all feeding rates followed by fish waste, palm oil waste and yard waste (Fig. 2). The level of food waste was significantly reduced compared to palm oil waste, fish waste and yard waste. The feeding rate of 0.50 g larva^−1^ day^−1^ for food waste showed the highest WRI followed by a feeding rate of 0.25 g larva^−1^ day^−1^ for fish waste and 1.00 g per larva per day for palm oil waste. The lowest WRI was recorded from yard waste at all feeding rates. For WRI, the interaction of feeding rate and four types of waste was significantly different (F = 36.86, *p* < 0.05).

## DISCUSSION

This study showed that food waste with a feeding rate of 1.00 g larva^−1^ day^−1^ produces the highest larval weight within the shortest development time. Food waste used in this experiment consists of a mixture of rice, vegetables, egg and meat, which is highly heterogeneous. The result of this experiment coincides with a study by Bonso (2013), in which a daily feeding rate of 1.00 g of food waste larva^−1^ day^−1^ produced better pre-pupae yield. In contrast, palm oil waste, fish waste and yard waste with a feeding rate of 1.50 g larva^−1^ day^−1^ produced the highest larvae/pre-pupae weight. In one study conducted by Leong *et al. (*2016), weight gained by BSF larvae increased as the feed amount of palm oil waste increased from 1 g day^−1^ to 25 g day^−1^. This matched with the result of this experiment as the feed amount of palm oil waste increased, the weight of BSF larvae also increased. Fish waste and yard waste used in this experiment comprised of mackerel fish’s head and dry mangoes leaves, respectively. The results of fish waste and yard waste can be compared with fish offal (St-Hilaire *et al*. 2007) which has high fat and protein content and rice straw (Manurung *et al*. 2016) which has high fibre content. Both studies showed that when the feed amount increased, the pre-pupae weight increased as well. In contrast, lower feeding rates employed in this study such as 0.25 g larva^−1^ day^−1^ and 0.50 g larva^−1^ day^−1^ resulted in comparatively low average larvae weights compared to the higher feeding rate mentioned earlier. This can be due to intraspecific competition that caused starvation and restricted development (Mitchell-Foster *et al*. 2012). From the lifecycle view, additional daily food supply shortened the development time and increased the pre-pupae weight.

However, BSF larvae which fed on food waste, palm oil waste and fish waste with a feeding rate of 2.00 g larva^−1^ day^−1^ revealed a lower prepupae weight but faster development time compared to that of feeding rate of 1.50 g larva^−1^ day^−1^. One possible explanation for the faster development time but lower pre-pupae weight gained by BSF is all three wastes of 2.00 g larva^−1^ day^−1^ feeding rate had a more compact medium compared to other feeding rates. In this experiment, none of the rearing substrates used were mechanically compacted during the period of the experiment. However, each feeding rate had a different compaction density. Holmes *et al*. (2013) suggested that high-density substrates are less penetrable subsequently affecting the feeding process. It is concluded that under an unfavourable condition such as a feeding rate of 2.00 g larva^−1^ day^−1^ with high compaction density, BSF larvae will feed and migrate out as soon as they had achieved minimum energy to perform pupal development (Diener *et al*. 2009), thus having a faster development time. In contrast, BSF larvae which fed on yard waste had a development time of 56 days for all feeding rates. As the development time of BSF larvae in yard waste exceeded the normal BSF larvae development time, the experiment ceased on day-56 and at that time only 10% to 15% of pre-pupae had emerged in all feeding rates. It was believed that yard waste does not have enough nutrients to support the growth of BSF larvae. This led to an increase in feeding duration to compensate for nutrient deficiency (Supriyatna *et al*. 2018). Yard waste media had an appropriate texture for BSF larvae, but the more significant factor could be dietary imbalance, mainly high-fibre content, and low content of some minerals. Although BSF larvae can degrade cellulose, hemicellulose and lignin, the high-fibre content in the BSF larvae feeding media reduced BSF larvae growth, survival, and development time (Rehman, Cai *et al*. 2017). The moisture content of yard waste was considered less than other waste media used in this study. When the larvae were exposed to dry conditions, the waste substrates solidify and difficult for BSF larvae to burrow and consume the substrate. As a result, the BSF larvae may cease feeding and prolong their development at this stage. Substrate moisture content is also an essential variable in the BSF bioconversion process. Feeding substrates with a 50% to 80% moisture level are ideal for larval growth, final weight, feed conversion efficiency, and biomass production (Cheng *et al*. 2017; Palma *et al*. 2018; Chen *et al*. 2019). A previous study investigated the impacts of faecal sludge moisture content at 65%, 75% and 85% and found that moisture content has a substantial impact on the pre-pupal dry weight. The greatest pre-pupae weight was achieved at 85% moisture content (Banks 2014). Lalander *et al*. (2020) found that BSF larvae reared at 75% to 88% moisture content had higher survival rates and body weight than those raised at 90% to 97.5% moisture content. The moisture content of the substrate determines its texture and controls larval motility, feed consumption, growth, and mortality (Makkar *et al*. 2014, Palma *et al*. 2018).

The larval mortality of BSF larvae that fed on palm oil waste, food waste and fish waste ranged from 1% to 4% which is considerably low. The low mortality rate of BSF larvae is probably due to the suitable moisture content of the feed substrate. The growth of BSF larvae is greatly influenced by the moisture content of the waste media (Cheng *et al*. 2017). Barragan-Fonseca *et al*. (2017) stated that feed with a moisture content of 52%–70% is adequate for the survival of BSF larvae. The moisture level of each waste is not measured in this experiment but in a study carried out by Leong *et al*. (2015), palm oil decanter cake had a moisture content of 63.13 ± 0.79%. Nevertheless, yard waste has an average larval mortality rate of up to 11% with the feeding rate of 1.50 g larva^−1^ day^−1^ having the highest mortality, 14%. This might be caused by the rapid moisture loss of yard waste. Yard waste has a finer texture and higher compaction density compared to other wastes. A study done by Holmes *et al. (*2013) found that potting soil which has a different texture to yard waste appears less compact and can retain more moisture than wood shavings.

For pre-pupae weight, the results obtained were comparable to previous studies by Cheng *et al*. (2017) and Meneguz *et al*. (2018). In contrast, Diener *et al*. (2009) applied five different daily food rates such as 12.5 mg, 25 mg, 50 mg, 100 mg and 200 mg larva^−1^ day^−1^ using the chicken feed. They reported that the amount of 100 mg larva^−1^ day^−1^ was the ideal feeding rate for the larvae while additional food supply did not reduce the development time of this species. However, the feeding rate of 1.00 g larva^−1^ day^−1^ used in this study showed 12 days development time compared to 16.6 days by their study. Other studies reported 15 days (Gobbi *et al*. 2013) and 21 days to 37 days (Oonincx *et al*. 2015) of development time after feeding the BSF larvae on chicken feed. The mortality rate is less than 4% and 2.3% for larvae reared in palm oil waste and food waste, respectively. This result was comparable with the 91%–99% survival rate recorded by Rehman, Rehman *et al*. (2017). Based on this study, we suggested that the feeding rates used in this study did not significantly affect the larval growth performance but potentially reduced the waste reduction efficiency. Furthermore, a reasonable pre-pupae wet weight was achieved after a shorter development time, showing great development with less mortality rate.

The higher WRI values reported in this study indicate the ability of BSF larvae to degrade different organic materials. This study showed that palm oil waste with a feeding rate of 1.00 g larva^−1^ day^−1^, fish waste with a feeding rate of 0.25 g larva^−1^ day^−1^ and food waste as well as yard waste with a feeding rate of 0.50 g larva^−1^ day^−1^ were the respective feeding rates for BSF larvae to greatly reduce the corresponding waste substrate. Increasing the daily feeding rate resulted in lower WRI values. A previous study conducted by Diener *et al*. (2009) showed the WRI value range from 1.1–3.8 g^−1^ day^−1^. A similar range of WRI values was also obtained in this experiment for food waste, palm oil waste and fish waste.

For palm oil waste, the WRI value obtained coincided with the study conducted by Leong *et al*. (2016). The WRI value increased as the feed amount of palm oil decanter cake increased from 1 g day^−1^ to 25 g day^−1^ (Leong *et al*. 2016). For food waste, WRI resulted from the feeding rates of 0.25 g and 0.50 g larva^−1^ day^−1^ do not differ significantly but the values deviate from the study of Bonso (2013) where 1 g larva^−1^ day^−1^ is the most suitable feeding rate to efficiently reduce organic waste. This dissimilarity might be due to the composition of the food waste used. Food waste used in Bonso (2013) study was a mix of cooked and uncooked food waste in a 1:1 ratio while the food waste used in this experiment was cooked food waste. The processing or cooked food usually involved heat treatment, crushing and changes of the substrate into a structure that promotes BSF larvae digestion (Pastor *et al*. 2015). Pre-treatment methods such as combination waste substrates and microbial fermentation enable BSF larvae to consume unsuitable nutrient-imbalanced and fibrous wastes (Isibika *et al*. 2019; Liu, Wang, *et al*. 2021). Heat pre-treatment causes fibre disintegration, causing it soft and easier to digest. However, prolonged exposure to high temperatures changes the chemical composition of various nutrients and depletes some nutritional qualities. Several studies on these strategies have been undertaken in an attempt to optimise the biodegradability of the waste substrate and facilitate the conversion process by BSF larvae (Zheng *et al*. 2012; Yu *et al*. 2011; Li *et al*. 2015; Rehman, Rehman *et al*. 2017; Isibika *et al*. 2019).

For fish waste and yard waste, the trend of WRI was similar to the WRI value conducted by Manurung *et al*. (2016), where the larvae provided with the lowest feeding rate had the highest WRI values. For yard waste, the WRI values range between 0.41 ± 0.02 to 0.71 ± 0.01 g day^−1^, comparable with the results obtained by Wong *et al*. (2020) using fermented coconut waste. The reduction observed in plant origin material like yard waste can be attributed to the ability of BSF larvae to secrete enzymes and harbour microbes that degrade yard waste (Gold *et al*. 2018). The amount of waste residue is then reduced because of this (Permana & Ramadhani 2018). In addition, due to their strong mouthparts and digestive enzymes, BSF larvae can consume large quantities of raw materials rapidly and efficiently than any other dipteran flies (Sheppard *et al*. 2002, Tomberlin *et al*. 2002). The digestive enzymes of BSF larvae have a biochemical base that comprises amylase, lipase, protease, and trypsin-like protease from the salivary gland and the intestine. Kim *et al*. (2011) investigated the enzyme activities and discovered that the activities of certain enzymes were considerably greater in BSF larvae than in house fly larvae. According to Ao *et al. (*2021), the microbial populations in rearing media contribute to substrate degradation. Bacterial genes that produce enzymes such as proteases, cellulases and lipases in the intestine of BSF larvae could digest cellulose, proteins and lipids. Thus, assisting in the breakdown of waste and other accumulated resources in this species. (Jeon *et al*. 2011; Lee *et al*. 2014).

The highest WRI values were recorded at 2.83 ± 0.10 and 2.87 ± 0.04 when larvae were fed with fruit waste and palm oil decanter cake, respectively (Leong *et al*. 2016). The WRI value obtained by Meneguz *et al*. (2018) also resembles this study for food waste and fish waste. The WRI for agro-industrial by-products (winery and brewery) and organic wastes (vegetables and fruits) were recorded at 2.4, 5.3 and 3.2, respectively. A low WRI indicates the wastage of the substrate either because it is not consumed or because it is in excess while a high WRI indicates possible starvation of the larvae at the feeding rate employed (Mutafela 2015). Food waste is rich in carbohydrates, fats, and proteins (Mutafela 2015) which enhance the conversion process by BSF larvae. This indicates palm oil waste has lower nutrient content than food waste (Rachmawati *et al*. 2010; Zheng *et al*. 2012) but higher nutrient content than yard waste. On the other hand, the low WRI values of fish waste might be linked to limited digestion due to soft fish bones, whilst palm oil waste and yard waste could be associated with high lignin-cellulose concentration (Oonincx *et al*. 2015; Manurung *et al*. 2016; Rehman, Cai *et al*. (2017)). Previous studies revealed that microorganisms in the intestine of the larvae could generate cellulase for cellulose digestion (Lee *et al*. 2014; Kim *et al*. 2011). The enzymes or microorganisms derived from BSF larvae could promote lignocellulose digestion and the conversion process of the waste substrate (Wu *et al*. 2015; Yu *et al*. 2011). More than half of the cellulose and hemicellulose could be transformed when a combination of enzymes and bacteria was added into the conversion system (Zheng *et al*. 2012). Microbial fermentation is a microbial modification method that increases the nutritional content of BSF larval feeding substrates (Raksasat *et al*. 2020). Thus, microbial fermentation can alter the nutritional and biochemical quality of feeding substrates, resulting in the increased development of BSF larvae. Anaerobic fermentation of waste media may also promote larval growth (Manurung *et al*. 2016). Furthermore, according to Mohd-Noor *et al. (*2016), self-fermented coconut waste (single substrate) had the highest waste reduction rate when compared to other mixed waste substrates. Therefore, the introduction of fermentation helps to break down the biopolymers and subsequently ease the bioconversion process by BSF larvae.

Due to its nutritional profile (40% crude protein and 33% crude fat) as determined by Nyakeri *et al*. (2017a), BSF larvae could serve as a sustainable and cheap potential protein source to be used by fish and poultry farmers. The high fat and calorie content in food waste was favoured by BSF larvae to develop their biomass (Nguyen *et al*. 2015). Food quality and nutrition intake affect the adult size, survival rate, and fertility while feeding rate is a crucial factor affecting the size and development of BSF larvae (Gobbi *et al*. 2013; Cheng *et al*. 2017). BSF larvae consume waste substrates for their growth and convert them into larvae nutritional compositions. This conversion process leads to the production of larval biomass and is further utilised to increase the value of industrial byproducts. The feeding rate and the resulting growth of BSF larvae are greatly important because the production of well-raised BSF larvae biomass is the requirement for high-quality animal feed, biodiesel, frass, and other products (Gao *et al*. 2019; Wong *et al*. 2020).

A study by Lalander *et al*. (2019) found that there is a strong correlation between volatile solid feeding rate and the final pre-pupae weight. Percentage of total volatile solid of food waste and palm oil waste were not measured in this experiment but in studies carried out by Lalander *et al*. (2019) and Kaosol and Rungarunanotai (2016), food waste and palm oil decanter cake had a total volatile solid value of 89.8 ± 4.0% and 87.3 ± 0.4% respectively. BSF larvae fed on fish waste had the fastest development time (11 days), but an overall lowest pre-pupae weight. This could be attributed to high levels of fat which have been found to be detrimental to BSF larval growth (Memon 2010). On the other hand, the longest larval development time of BSF larvae reared on yard waste could be attributed to low nutritional quality and high levels of lignin and cellulose as earlier explained (Li *et al*. 2011).

## CONCLUSION

Higher feeding rates of waste enhanced growth performance but notably had an adverse impact on waste reduction. It is observed that the BSF larvae reduced more waste effectively with a lower feeding rate. Although this study documented no detrimental effects on the growth performance of BSF larvae, higher feeding rates are not recommended because they can cause inappropriate management of the substrate. To save cost and resources, an ideal feeding rate should be implemented for a greater bioconversion process. Ultimately, this study provides valuable insights into waste management strategies in developing countries. The effects of feeding rate, development time and waste reduction efficiency are greatly important when employing BSF bioconversion for organic waste recycling. An understanding of insect growth and waste reduction efficiency is needed to make this biological process more feasible.

## Figures and Tables

**Table 1:** BSF larvae growth and waste reduction efficiency of food waste.

Food waste	0.25 g		0.50 g		1.00 g		1.50 g		2.00 g	
				
	Mean	SE	Mean	SE	Mean	SE	Mean	SE	Mean	SE
Development time (larvae to pre-pupae, days)	20.00	0.00	14.00	0.00	12.00	0.00	12.00	0.00	11.00	0.00
Larval mortality (%)	2.33^a^	0.33	1.33^a^	0.67	0.67^a^	0.67	0.67^a^	0.67	2.33^a^	0.33
Pre-pupae wet weight (g)	11.22^a^	0.03	12.03^a,b^	0.33	12.73^b^	0.24	12.28^a,b^	0.28	12.34^a,b^	0.29
WRI	4.23^a^	0.05	4.43^a^	0.06	3.14^b^	0.07	2.72^c^	0.05	2.64^c^	0.02

*Note*:

* Mean values followed by the same letter in the same row do not vary significantly (*p* < 0.05).

**Table 2:** BSF larvae growth and waste reduction efficiency of palm oil waste.

Palm oil waste	0.25 g		0.50 g		1.00 g		1.50 g		2.00 g	
				
	Mean	SE	Mean	SE	Mean	SE	Mean	SE	Mean	SE
Development time (larvae to pre-pupae, days)	19.00	0.00	16.00	0.00	13.00	0.00	13.00	0.00	12.00	0.00
Larval mortality (%)	4.00^a^	0.00	3.00^a^	0.58	2.67^a^	1.53	2.67^a^	1.53	3.00^a^	1.00
Pre-pupae wet weight (g)	5.96^a^	0.12	8.34^b^	0.15	10.70^c^	0.17	11.90^d^	0.38	8.30^b^	0.27
WRI	1.68^a^	0.06	1.76^a,b^	0.05	1.89^b^	0.02	1.39^c^	0.01	1.08^d^	0.01

*Note*:

* Mean values followed by the same letter in the same row do not vary significantly (*p* < 0.05).

**Table 3:** BSF larvae growth and waste reduction efficiency of fish waste.

Fish waste	0.25 g		0.50 g		1.00 g		1.50 g		2.00 g	
				
	Mean	SE	Mean	SE	Mean	SE	Mean	SE	Mean	SE
Development time (larvae to pre-pupae, days)	16.00	0.00	14.00	0.00	12.00	0.00	12.00	0.00	10.00	0.00
Larval mortality (%)	0.67^a^	0.33	0.67^a^	0.67	2.00^a^	0.58	1.33^a^	0.88	2.67^a^	0.33
Pre-pupae wet weight (g)	4.29^a^	0.02	4.72^b^	0.04	5.34^c^	0.01	5.69^d^	0.06	5.22^c^	0.00
WRI	3.75^a^	0.24	2.75^b^	0.09	2.03^c^	0.05	1.92^c^	0.14	1.55^c^	0.03

*Note*:

* Mean values followed by the same letter in the same row do not vary significantly (*p* < 0.05).

**Table 4:** BSF larvae growth and waste reduction efficiency of yard waste.

Yard waste	0.25 g		0.50 g		1.00 g		1.50 g		2.00 g	
				
	Mean	SE	Mean	SE	Mean	SE	Mean	SE	Mean	SE
Development time (larvae to pre-pupae, days)	56.00	0.00	56.00	0.00	56.00	0.00	56.00	0.00	56.00	0.00
Larval mortality (%)	12.67^a^	0.88	8.67^b^	0.88	6.33^b^	0.88	14.00^a^	0.58	12.67^a^	0.67
Larvae/pre-pupae wet weight (g)	3.39^a^	0.13	5.09^b^	0.18	6.98^c^	0.12	7.78^c^	0.14	7.19^c^	0.25
WRI	0.53^a,b^	0.02	0.71^b^	0.01	0.41^a^	0.02	0.44^a^	0.01	0.65^a,b^	0.12

*Notes*: Only 10%–15% of the larvae change into pre-pupae in yard waste. Mean values followed by the same letter in the same row do not vary significantly (*p* < 0.05).
